# Sarcoptic Mange of Fox Origin in Multiple Farm Animals and Scabies in Humans, Switzerland, 2018

**DOI:** 10.3201/eid2506.181891

**Published:** 2019-06

**Authors:** Simone R.R. Pisano, Marie-Pierre Ryser-Degiorgis, Luca Rossi, Andrea Peano, Karin Keckeis, Petra Roosje

**Affiliations:** University of Bern, Bern, Switzerland (S.R.R. Pisano, M.-P. Ryser-Degiorgis, P. Roosje);; University of Turin, Grugliasco, Italy (L. Rossi, A. Peano);; consulting veterinarian, Wabern, Switzerland (K. Keckeis)

**Keywords:** *Sarcoptes scabiei*, mites, sarcoptic mange, scabies, zoonoses, wildlife, livestock, red fox, *Vulpes vulpes*, farm animals, Jura Mountains, Switzerland, skin lesions, outbreak, vector-borne infections

## Abstract

Fox-derived *Sarcoptes scabiei* mites caused an outbreak of mange on a farm in Switzerland in 2018. Pruritic skin lesions suggestive of *S. scabiei* mite infestation developed in 4 humans who had direct contact with affected farm animals but not foxes. Sarcoptic mange is continuously spreading; such outbreaks affecting humans could start occurring more frequently.

The *Sarcoptes scabiei* mite is the causative agent of scabies in humans and sarcoptic mange in animals (*1*). Scabies is considered a neglected reemerging disease of public health concern (*2*). Sarcoptic mange causes distress in livestock, economic loss in the livestock industry, and disease and death in wildlife (*3*). The degrees of host specificity and cross-infectivity of *S. scabiei* mites are still debated (*3*).

In January 2018, sarcoptic mange was suspected on a farm in the Jura Mountains, Switzerland. The outdoor loose housing system of this farm hosting 2 oxen (*Bos taurus*), 2 horses (*Equus caballus*), 5 goats (*Capra hircus*), 4 alpacas (*Vicugna pacos*), 8 fallow deer (*Dama dama*), and 15 sheep (*Ovis aries*) was separated from a stable housing 3 pigs (*Sus scrofa domesticus*). Six dogs (*Canis lupus familiaris*) and 17 cats (*Felis catus*) had access to all stables. Pruritic skin lesions developed in several species 2–3 weeks after repeated episodes of mangy red foxes (*Vulpes vulpes*) sleeping in the stables and making partial body contact with the livestock (Figure, panels A, B). Pruritic skin lesions also developed in 4 persons who had direct contact with the domestic animals but not the foxes. A fox with mange was found dead nearby and a necropsy was performed. Oxen, dogs, and pigs were treated with avermectins before diagnostic investigations were carried out.

**Figure:**
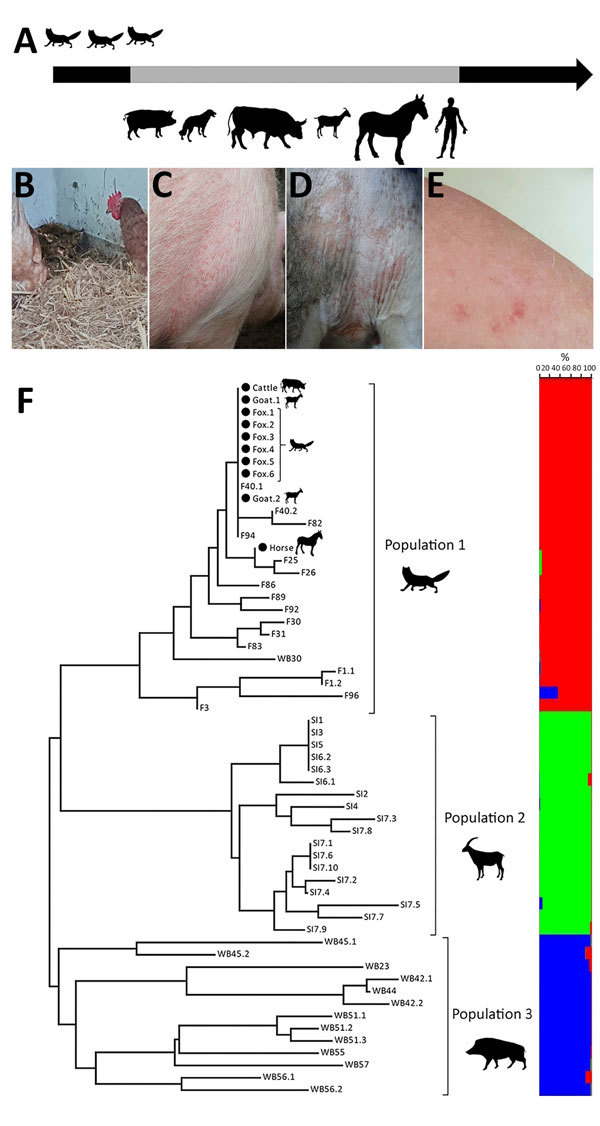
Clinical and molecular characterization of an outbreak of fox-derived *Sarcoptes scabiei* mites in multiple mammal species on a farm in Switzerland, 2018. A) Outbreak timeline displaying animal species (pigs [*Sus scrofa domesticus*], oxen [*Bos taurus*], dogs [*Canis lupus familiaris*], goats [*Capra hircus*], horses [*Equus caballus*], and red foxes [*Vulpes vulpes*]) showing clinical signs compatible with sarcoptic mange and humans with signs of zoonotic scabies in order of appearance. Gray portion of arrow indicates the period during which clinical signs were observed in domestic animals and humans. Foxes with mange were observed in stables up to 3 weeks before the beginning of clinical signs in livestock. B–E) Clinical signs observed in a red fox (B; lethargy, severe hyperkeratosis), a pig (C; erythematous papules on shoulder and thorax), an ox (D; alopecia and erythema in the perineal region), and a teenage girl (E; erythematous papules on an arm). F) Multilocus microsatellite analysis demonstrating the genetic relationship of 10 individual mites isolated from a horse, an ox, a goat, and a fox at the farm where the outbreak occurred (black dots) and 48 additional mites from red foxes from the same region of Switzerland (population 1); Iberian ibex (*Capra pyrenaica*) from southern Spain (population 2); and wild boars (*Sus scrofa*) from Switzerland, nearby areas of France, and northern Italy (population 3). Neighbor-joining tree (left) constructed by using distance matrices with Populations version 1.2.28 (http://bioinformatics.org/populations) and displayed by using MEGA4 (http://www.megasoftware.net). Tree branch lengths are proportional to the percent genetic distance. Bar plot (right) obtained with Structure 2.3.4 (https://web.stanford.edu/group/pritchardlab/structure.html) represents the cluster membership according to the analyses of 9 markers for K = 3 with the probability (0%–100%) for each mite to belong to a different population. Red indicates population 1, green indicates population 2, and blue indicates population 3. The 3 populations are the same as those in the distance tree. F, red fox; SI, Iberian ibex; WB, wild boar.

Clinical examination revealed papules, erythema, excoriations, hyperkeratosis, and hypotrichosis with variable severity in 2 pigs, 2 goats, 2 dogs, all horses, and all oxen (Figure, panels C, D). The 3 sheep and 1 cat examined did not have lesions suggestive of mange. Close examination of the fallow deer and alpacas was impracticable. Humans had pruritic erythematous papules and excoriations on their neck, legs, or arms (Figure, panel E). Health authorities temporarily prohibited 1 affected person (a teenager) from attending school because of suspected scabies. Pruritus and skin lesions disappeared in the affected animals and humans within 6 weeks after >2 treatments with avermectins, topical neem oil, or both.

We identified *S. scabiei* mites by light microscopy in the skin scrapings from 2 pigs, 1 horse, 1 ox, 1 goat, and 1 fox but none of the scrapings from 3 sheep, 5 dogs, and 1 cat sampled. A few mites but no eggs, eggshells, or gravid females were observed on livestock, whereas all stages were present and numerous on the fox. Skin scrapings were not obtained from the affected humans. We confirmed *S. scabiei* mite mitochondrial 16S rDNA by TaqMan real-time PCR (*4*) in the sampled horse, ox, goat, and fox but not in the sampled sheep, cat, dogs, or pigs. Analyses with a panel of 9 microsatellites (sarms 33, 35–38, 40, 41, 44, and 45) (*5*) confirmed that foxes were the source of the mites (Figure, panel F); the mites on the outbreak farm were similar to each other and to mites previously collected from foxes in Switzerland (*6*) but different from those collected from wild ungulates in Spain, Switzerland, France, and Italy (*5*,*7*,*8*).

Genetic investigations suggest that multiple *S. scabiei* mite subpopulations can infect the same host and that mite subpopulations can differ from host to host. Different subpopulations undergo varying degrees of gene flow depending on the geographic distances among infested hosts and cluster in animals that share a taxonomic classification above the species level (*1**,**9*). In Europe, wildlife herbivore-, carnivore- and omnivore-derived *S. scabiei* mites have been described as distinct groups, and intraspecies and interspecies transmission have been proposed to occur among hosts of the same taxon but not among different taxa under natural conditions (*8*). However, prey-to-predator transmission was demonstrated in Africa (*10*). Thus, direct contact between affected hosts or fomites and susceptible hosts (*1*) rather than mite host specificity might determine whether *S. scabiei* mites are transmitted to different taxonomic groups.

Our investigation unambiguously identified wild carnivore–derived *S. scabiei* mites as the cause of a point-like outbreak involving different domestic herbivores and omnivores. However, we found no evidence of mite reproduction, which suggests that the mites that transmitted from foxes to other species were not able to actively replicate. Yet, persistence of clinical signs despite treatment and suspected subsequent transmission from domestic animals to humans is not fully consistent with the self-limiting pattern described for zoonotic scabies, although reinfection of domestic animals by other foxes with mange could have occurred.

Increased fox abundance, reemergence, and continuous spread of sarcoptic mange in foxes could lead to its emergence in other wild and domestic animal species. Although mites or their DNA could not be demonstrated in the affected humans, their clinical signs were highly suggestive of scabies, highlighting the zoonotic potential of *S. scabiei* mites. The propensity of foxes with mange to live close to human settlements, the increase in green farming, and increased density and size of domestic animal populations augment the risk for contacts between foxes, domestic animals, and humans. Therefore, such outbreaks might become more frequent in the future.
